# An unusual association between hemophagocytic lymphohistiocytosis, mixed connective tissue disease, and autoimmune hemolytic anemia: A case report

**DOI:** 10.1097/MD.0000000000007488

**Published:** 2017-07-14

**Authors:** Amar H. Kelkar, Anushi A. Shah, Sherri L. Yong, Zohair Ahmed

**Affiliations:** a Department of Internal Medicine; b Department of Pathology, University of Illinois College of Medicine at Peoria, Peoria; c Department of Gastroenterology, University of Illinois College of Medicine at Chicago, Chicago, IL.

**Keywords:** autoimmune hemolytic anemia, hemophagocytic lymphohistiocytosis, HLH, mixed connective tissue disorder, nephrotic syndrome

## Abstract

**Rationale:**

In the adult patient, hemophagocytic lymphohistiocytosis (HLH) is uncommon and frequently difficult to diagnose due to its nonspecific presentation and numerous complications.

**Patient concerns:**

Herein, we present the case of a 25-year-old female who initially presented for evaluation of persistent fevers and fatigue. She was found to have splenomegaly, generalized lymphadenopathy, pancytopenia, and acute hepatic failure.

**Diagnoses, Interventions, and Outcomes:**

Her course was further complicated by the development of nephrotic syndrome and autoimmune hemolytic anemia (AIHA). Antinuclear antibody and ribonucleoprotein were positive, with concurrent physical examination findings, indicating underlying mixed connective tissue disease (MCTD). Ferritin was greater than 40,000 ng/dL. Viral studies, including hepatitis A, B, and C, cytomegalovirus, and Epstein–Barr virus were negative. On the basis of her clinical presentation, a diagnosis of HLH secondary to MCTD was made. This was later confirmed on liver biopsy. She was started on high-dose prednisone and her symptoms completely resolved. She was then transitioned to azathioprine, hydroxychloroquine, prophylactic antibiotics, and a prednisone taper for long-term management.

**Lessons:**

This case is notable for the association of both AIHA and MCTD with HLH, providing support for a possible relationship between these 3 conditions.

## 1 Introduction

First diagnosed in 1939 as histiocytic medullary reticulocytosis, hemophagocytic lymphohistiocytosis (HLH) is a rare autoimmune disorder that is usually associated with the pediatric population. It has also been identified as macrophage activation syndrome when associated with connective tissue diseases. HLH can rapidly progress to multiorgan failure and death, thus early diagnosis and treatment are essential to survival.^[1,2]^

There are 2 main classifications of HLH. Primary (or familial) HLH is hereditary with an autosomal recessive pattern and typically presents at a very young age. Secondary (sporadic) HLH occurs in response to an inciting stimulus and has been associated with heterozygous germline mutations. It has no age predominance. Epstein–Barr virus and lymphoma are most commonly associated; however, other infectious, rheumatologic, and neoplastic etiologies have been identified.^[1,2]^

On the basis of the initial presentation of acute hepatic failure, complications including nephrotic syndrome and warm autoimmune hemolytic anemia (AIHA), and the underlying etiology of mixed connective tissue disease (MCTD), this case represents an atypical presentation of this rare disorder in an adult. We hope this case report will assist in early identification of HLH and serve as a testimonial toward a possible relationship between HLH, AIHA, and MCTD.^[1]^

## 2 Case history and clinical course

A 25-year-old African–American female with a past medical history of eczema and herpes zoster presented to the emergency department with a 6-day history of recurrent fevers, night sweats, and lethargy. Her social history was negative for high-risk sexual behavior, ethanol abuse, or intravenous drug use. Her family history was noncontributory. Physical examination revealed a temperature of 39.5°C, splenomegaly, and diffuse nontender lymphadenopathy involving cervical, posterior occipital, axillary, and inguinal lymph nodes. Presenting laboratory data revealed leukopenia (1.43 X 10^3 cells/μL), neutropenia (0.29 X 10^3 cells/μL), thrombocytopenia (78 X 10^3 platelets/μL), hyponatremia (133 millimoles/L), hypokalemia (2.7 millimoles/L), and abnormal liver function studies with aspartate aminotransferase (AST) 337 units/L, alanine aminotransferase (ALT) 185 units/L, total bilirubin 1.8 mg/dL, normal alkaline phosphatase, and elevated international normalized ratio (INR) of 1.5. Both erythrocyte sedimentation rate and C-reactive protein levels were normal. She was empirically started on cefepime for febrile neutropenia. After admission, her liver function acutely deteriorated with AST 3629 units/L, ALT 1647 units/L, and INR 2.0. The platelet count decreased to 45 X 10^3 platelets/μL. Her ferritin level was greater than 40,000 nmilliliter/mL. Ceruloplasmin, ammonia, ethanol, and acetaminophen levels were within normal limits. Blood cultures were negative. Viral workup, by IgM antibody and viral titers, was negative for human immunodeficiency virus, hepatitis A, B, and C, herpes simplex virus, herpes zoster, parvovirus, Epstein–Barr virus, cytomegalovirus, and adenovirus. An initial autoimmune workup revealed an antinuclear antibody titer 1 : 160 with a speckled pattern. Subsequent extractable nuclear antigen panel was positive for smooth muscle ribonucleoprotein antigens (greater than 200 extractable units). Anti-mitochondrial antibody, anti-liver kidney microsomal type 1 antibody, and anti-smooth muscle antibody were normal. Doppler ultrasound of her abdomen showed normal bile ducts and a patent portal vein. Computed tomography of the abdomen confirmed moderate splenomegaly. Over the course of the hospitalization, the patient developed facial erythroderma, swelling of both hands, Raynaud phenomenon, muscle tenderness, and respiratory insufficiency. Jaundice and pruritus also developed, in correlation with worsening hyperbilirubinemia. Total bilirubin level peaked at 21.9 mg/dL with corresponding direct bilirubin 14.3 mg/dL. Hemoglobin levels dropped to 7.6 g/dL with corresponding haptoglobin levels less than 8 mg/dL and lactate dehydrogenase 1170 units/L. The direct antiglobulin test was positive for anti-IgG antibodies and negative for complement C3d, confirming warm AIHA (Table 1). Serial peripheral blood smears showed only rare schistocytes and serum protein electrophoresis was negative for hemoglobinopathy. Although she had relatively low fibrinogen levels (152 mg/dL), suspicion for clinically significant disseminated intravascular coagulation was low. She subsequently developed nephrotic-range proteinuria (greater than 5 g over 24 hours) with normal serum creatinine levels.^[3]^ After consultations from hematology, nephrology, and rheumatology, the patient was started on 80 mg of oral prednisone daily for warm AIHA with gradual clinical improvement. She underwent biopsies of both liver and kidney. Simultaneously, a more extensive autoimmune workup was ordered, which included complement testing, anti-double stranded DNA antibody, anti-neutrophil cytoplasmic antibodies, anti-cardiolipin antibody, anti-beta-2-glycoprotein I antibodies, lupus anticoagulant, anti-smith antibody, anti-histone antibody, cryoglobulin, ADAMTS13 activity, and creatine phosphokinase (CPK). The CPK level was elevated at 445 units/L, whereas the remainder of the workup was unremarkable. Fasting triglyceride level was also found to be elevated (331 mg/dL). Blood samples were sent to Cincinnati Children's Hospital, to assess natural killer (NK) cell function, and these showed low overall NK cell population and elevated soluble CD163 (2127 n/mL). Soluble interleukin-2 (IL-2) receptor level, a marker of lymphocyte activity, was measured at 482 pg/mL.^[1]^

**Table 1:**
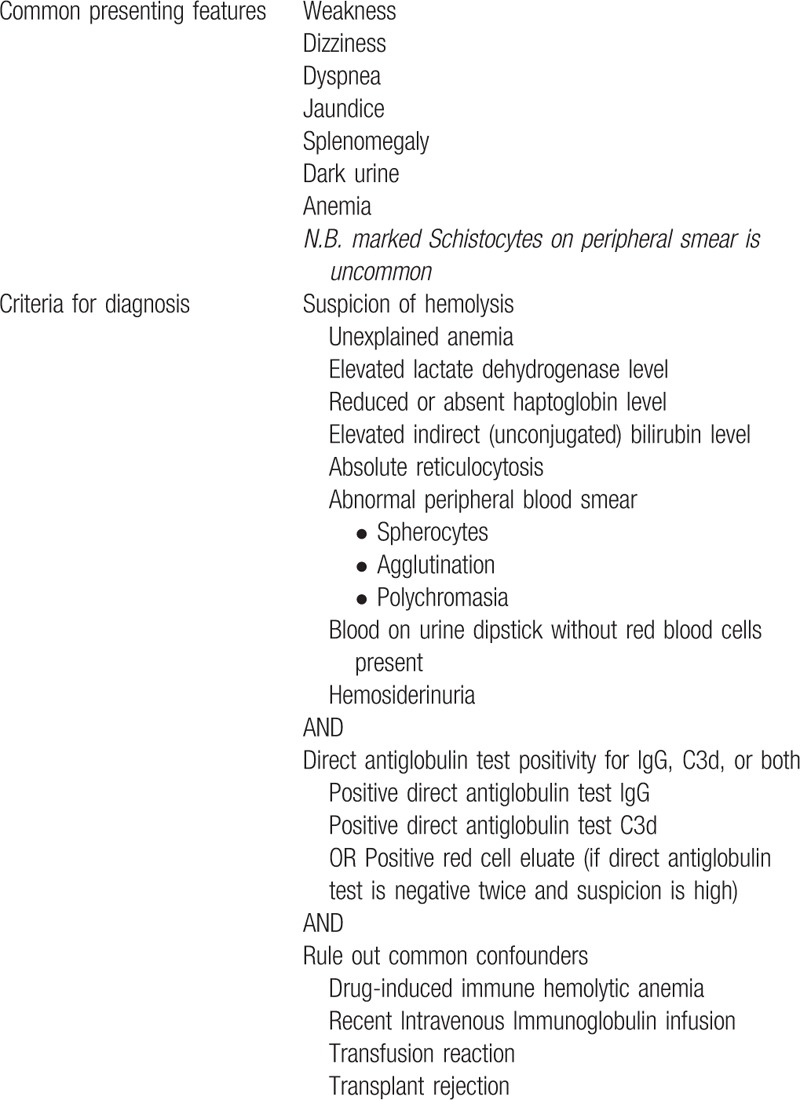
Autoimmune Hemolytic Anemia Diagnostic Criteria (based on British Society for Haematology guideline)^[3]^.

The steroid regimen was increased to 250 mg of pulsed intravenous solumedrol daily for 3 days while awaiting her biopsy results. This resulted in significant clinical recovery with resolution of her fevers, pancytopenia, elevated liver enzymes, coagulopathy, and proteinuria.

**Figure 1:**
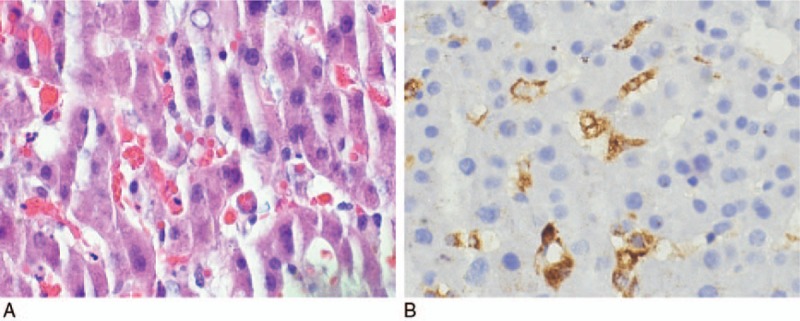
(A) Haemotoxylin and eosin stain of liver biopsy with Kupffer cell hyperplasia and hemophagocytosis. Kupffer cells are liver-specific macrophages. (B) CD68 positive stain of liver biopsy showing Kupffer cells, with negative staining of intracytoplasmic red blood cells, demonstrating hemophagocytosis. CD68 is an immunohistochemical marker used for macrophages. Kupffer cells are liver-specific macrophages.

Her kidney biopsy results were nonspecific and not consistent with glomerulonephritis. The liver biopsy revealed Kupffer cell hyperplasia associated with hemophagocytosis (Fig. 1A, B), confirming the diagnosis of HLH.

### 2.1 Diagnosis

Due to the nonspecific presentation of HLH, patients typically undergo lengthy workup, resulting in delayed diagnosis and treatment with high mortality from rapidly progressive multiorgan failure. To expedite identification of HLH, the Histiocyte Society created revised guidelines in 2004. A clinical diagnosis can be made in a patient who meets 5 of 8 of the following criteria: fever, splenomegaly, bicytopenia, hypertriglyceridemia or hypofibrogenemia, pathology confirming hemophagocytosis, elevated ferritin count, low NK cell activity, and high soluble IL-2 receptor levels (Table 2).^[1]^

**Table 2:**
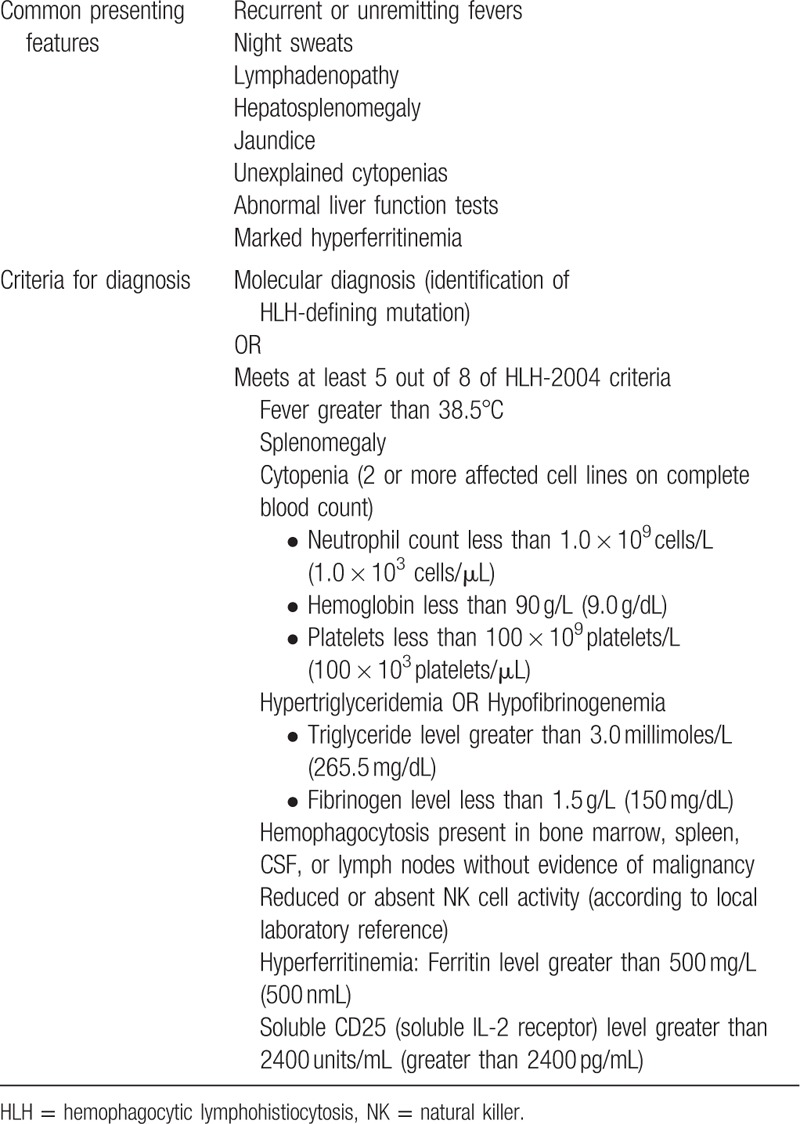
Hemophagocytic Lymphohistiocytosis Diagnostic Criteria (based on the HLH-2004 guideline)^[1]^.

In the case of our patient, achieving the diagnosis of HLH was a convoluted process and the differential diagnosis evolved as laboratory results became available.

Due to elevated transaminases and INR, the initial concern was for acute hepatic failure. The most common drug and viral causes were ruled out by laboratory testing. Autoimmune hepatitis was a significant consideration, but it was ruled out by biopsy, while simultaneously revealing hemophagocytosis. These findings in the setting of fevers, splenomegaly on examination and imaging, lymphadenopathy, pancytopenia, significant hyperferritinemia, hypertriglyceridemia, and hypofibrinogenemia met 6 of the 8 HLH-2004 guideline criteria, which was sufficient for clinical diagnosis of HLH. To further clarify the diagnosis, primary and secondary causes were evaluated. Primary HLH was largely ruled out on the basis of the patient's age and the laboratory findings from Cincinnati Children's Hospital. Of the secondary causes of HLH, common viral etiologies were ruled out. With largely normal peripheral blood smears, resolution of lymphadenopathy, and normal bone marrow biopsy findings after hospital discharge, hematological malignancies including lymphoma were also ruled out.^[1]^

Differential diagnoses for both nephrotic syndrome and warm AIHA were also considered. Additional workup was largely normal for both and the patient had no history of transfusions or transplant. Furthermore, drug-induced causes for both were considered unlikely on the basis of her short list of medications and the timeline of events. Thus, we suspected that both were complications of underlying connective tissue disease and HLH.^[3]^ On the basis of the elevated, speckled antinuclear antibody titer and smooth muscle ribonucleoprotein antigen, the patient met serological criteria for MCTD. The patient also had findings of hand swelling, Raynaud phenomenon, and myositis. Her presentation coincided closely with the criteria established by Alarcón–Segovia, allowing for a diagnosis of MCTD (Table 3).^[4]^

**Table 3:**
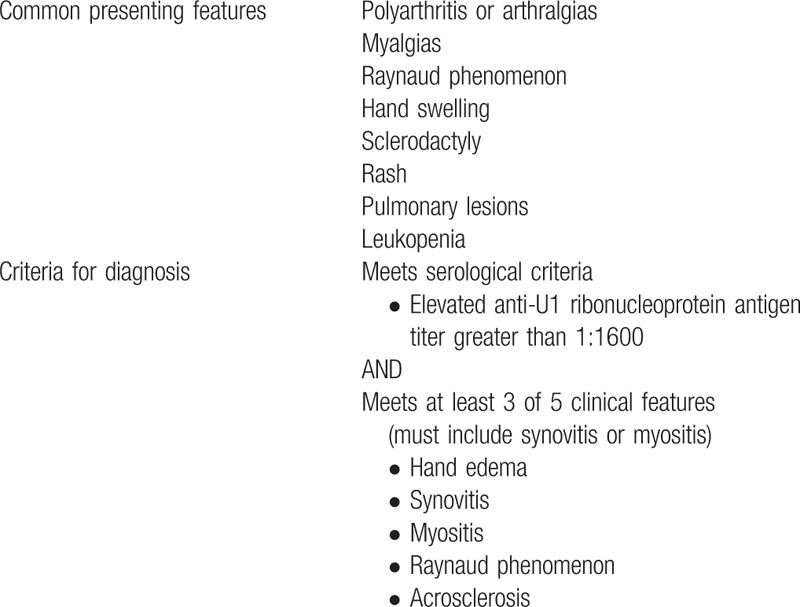
Mixed Connective Tissue Disease Diagnostic Criteria (based on criteria by Alarcón-Segovia)^[4]^.

Together, these findings supported a parsimonious diagnosis of HLH secondary to MCTD and complicated by warm AIHA and nephrotic syndrome.

## 3 Management

In the case of secondary HLH, treatment of the underlying trigger may be sufficient. The focus of HLH therapy is to reduce systemic inflammation by suppressing immune cells. The first treatment protocol was designed in 1994 by the Histiocyte Society. It consists of weekly dexamethasone and etoposide. In the case of neurological involvement, intrathecal methotrexate and hydrocortisone are recommended. Allogeneic hematopoietic stem cell transplant is the preferred curative therapy if sustained remission cannot be achieved by immunosuppressants.^[1]^

On the basis of our review of the existing literature, there was only one other documented case of HLH secondary to MCTD, and management required second-line therapy with intermittent cyclophosphamide pulse therapy due to corticosteroid-resistant HLH.^[5,6]^

Given our patient's initial response to steroids, we opted to treat her MCTD with 250 mg of pulsed intravenous solumedrol daily for 3 days followed by 80 mg of oral prednisone daily and 200 mg of oral hydroxychloroquine twice daily. Rapid improvement was seen with this regimen and induction with other immunosuppressants was initially spared.

## 4 Outcome and follow-up

The patient remains hemodynamically stable with continuing resolution of her clinical symptoms. Her liver enzymes are now within normal limits. The ferritin level has decreased from greater than 40,000 to 148 n/mL. Renal studies improved with her most recent urine protein-creatinine ratio being 0.85 mg/dL, which is down from 5.49 mg/dL. However, she has exhibited worsening lymphocytopenia with the CD4-positive T lymphocyte count being 22 cells/mL^3^.

After further evaluation in the outpatient setting, including bone marrow biopsy and flow cytometry, data were sufficient to rule out hematological malignancies. Azathioprine was started after this confirmation, along with antibiotic prophylaxis for opportunistic infections. Her other current medications include 200 mg of oral hydroxychloroquine twice daily and a prednisone taper.

## 5 Discussion

In cases of HLH, common presenting symptoms include unremitting fevers, lymphadenopathy, and cytopenias. Given this nonspecific presentation, many cases are associated with adverse outcomes due difficulty establishing the diagnosis, numerous complications, and subsequent delay in initiation of appropriate immunosuppressive therapies. As such, treatment should be strongly considered in cases with a high degree of suspicion. We hope this case will provide some insight into the recognition and management of HLH.^[1,6]^

HLH occurs due to disturbances in a common pathway utilized in cell-mediated immunity; however, due to low disease incidence, the molecular basis of its causes is not well-described in the literature. Normally, antigen-presenting cells (APCs) such as macrophages and their tissue derivative, histiocytes, are activated by cytokines, with regulatory mechanisms in place to limit their activity. In patients with HLH, one or more upstream pathogenic stimuli cause a cytokine storm and dysregulation of this pathway. This is characterized by marked inflammation and tissue destruction by these APCs, including phagocytosis of erythrocytes. Destruction of erythrocytes also leads to upregulation of soluble CD163, a hemoglobin scavenger receptor, which is a testable serum marker of HLH. This series of events is how both HLH and macrophage activation syndrome derive their names.^[1,2,7]^

Study of primary HLH has revealed several inheritable defects, including ones to lytic enzymes perforin and granzyme as well as to intracellular trafficking mechanisms affecting lytic granule migration in NK cells and CTLs. The potential downstream effects include failure to clear infected, infectious, or abnormal cells and expansion of these ineffective cytotoxic cell populations. These defective cells can activate the pathway described above by releasing cytokines, leading to the unregulated APC activity. In the case of our patient, serum samples sent to Cincinnati Children's Hospital were evaluated for NK function and lytic granule function, to rule out common causes of primary HLH.^[1,2,7]^

Although the mechanisms of secondary HLH have not been demonstrated to the same level of detail, it is believed that another trigger causes dysfunction in this same pathway. Hypothetically, destruction of normal immune cells, or unregulated cytokine release, related to a flare of underlying connective tissue diseases could serve as the trigger for disease expression. Given that our patient had an unusually small population of functionally normal NK cells, we hypothesize that a flare of MCTD led to suppression of her innate immune system, cytokine storm, and ultimately HLH. Further study of the disease processes has the potential to improve our understanding of immune regulatory mechanisms and improve treatment modalities.^[1,2,7]^

Although many patients with HLH seem to develop bizarre complications during their disease course due to the complex underlying pathophysiology of the disorder, there were several notable features in our patient's case that should be highlighted. Early liver dysfunction is very common in HLH, with as many as 60% of cases presenting with abnormal liver function testing. However, fewer cases progress to acute hepatic failure, as was the case in our patient. This may warrant future consideration as an important differential diagnosis in liver failure.^[1]^

The development of nephrotic syndrome is also a well-documented complication, seen in approximately 38% of cases. However, the development of AIHA distinguishes this case, as it has been rarely described in the literature in association with HLH. In those particular cases, HLH has simultaneously been associated with connective tissue disease or pregnancy. It was suspected that expression of our patient's underlying MCTD was the trigger for HLH, which fits this pattern. The association of HLH with MCTD is also quite rare, as there has only been one other case reported in the literature. Give this unusual set of circumstances, our case provides additional evidence toward a relationship between this triad of conditions that requires further study.^[1,5,6,8]^

## 6 Methods

Informed consent from the patient was requested and obtained from the patient. Ethical approval from our Institutional Review Board was not necessary, as this is a case report that maintains the anonymity of the patient.

## 7 Patient perspective

The patient was offered the option to share her written perspective, however, and she politely refused.

## Acknowledgment

The authors would like to thank our patient for allowing for her case to be presented.
